# Complete genome sequence of “*Candidatus* Phytoplasma pruni” PR2021, an uncultivated bacterium associated with poinsettia (*Euphorbia pulcherrima*)

**DOI:** 10.1128/MRA.00443-23

**Published:** 2023-07-18

**Authors:** Shen-Chian Pei, Ai-Ping Chen, Shu-Jen Chou, Ting-Hsuan Hung, Chih-Horng Kuo

**Affiliations:** 1 Institute of Plant and Microbial Biology, Academia Sinica, Taipei, Taiwan; 2 Department of Plant Pathology and Microbiology, National Taiwan University, Taipei, Taiwan; The University of Arizona, Tucson, Arizona, USA

**Keywords:** phytoplasma, genome, mollicutes, pathogen, plant pathogens

## Abstract

The complete genome sequence of “*Candidatus* Phytoplasma pruni” strain PR2021, which consists of one 705,138 bp circular chromosome and one 4,757 bp circular plasmid, is presented in this work. This bacterium is associated with poinsettia (*Euphorbia pulcherrima*) cultivar “Princettia Pink.”

## ANNOUNCEMENT

Phytoplasmas are bacteria that induce developmental abnormalities in plants (1). Although such abnormalities result in economic losses for most crops, the enhanced branching increases the ornamental value of poinsettia (2). Therefore, phytoplasmas have been integrated into the breeding of poinsettia cultivars. To investigate the key genes for functional characterization, we sequenced the genome of “*Candidatus* Phytoplasma pruni” PR2021 associated with cultivar “Princettia Pink.” All kits were used according to the manufacturer’s protocols, and all bioinformatics tools were used with the default settings unless stated otherwise.

The plant material was purchased from Dayi Agritech (Taipei, Taiwan), and midribs were collected for DNA extraction without cultivation using a cetyltrimethylammonium bromide method (3). The DNA sample was further processed using AMPure XP (Beckman Coulter, Brea, CA, USA), Short Read Eliminator XL Kit (PacBio, Menlo Park, CA, USA), and NEBNext Microbiome DNA Enrichment Kit (New England Biolabs, Ipswich, MA, USA) to enrich long fragments of bacterial DNA. For Illumina sequencing, the library was prepared using KAPA LTP Library Preparation Kit (Roche, Switzerland) and sequenced on a NovaSeq 6000 using the S4 Reagent Kit v1.5 to generate 67.9 Gb of 150 bp paired-end reads. Reads were trimmed with a Q20 cutoff and filtered using a 100-bp cutoff. For Oxford Nanopore Technologies (ONT) sequencing, the library was prepared using ONT Ligation Sequencing Kit (SQK-LSK114) without shearing and sequenced on a MinION (FLO-MIN114). Basecalling was performed using Guppy v6.4.2 with the high accuracy mode and a minimum *q*-score of 9. A total of 1,704,843 reads were generated, with a combined length of 27.9 Gb and a N50 of 23.4 kb.

The first *de novo* assembly was based on Velvet v1.2.10 (4) with only the Illumina reads. Putative phytoplasma contigs were identified by BLASTX (5) searches against phytoplasma proteins available in the NCBI-nr database (6). All raw reads were mapped to these contigs using BWA v0.7.17 (7) and Minimap2 v2.15 (8). The Illumina reads with an alignment score >30 and the ONT reads with an alignment score >1,000 were extracted for the second step of hybrid assembly using Unicycler v0.4.9b (9). Raw reads mapping and contig extension were repeated until all contigs became circular (i.e., ONT reads spanning both ends). The procedure of gene prediction and annotation was based on the one described in our previous work (10, 11). For gene prediction, RNAmmer v1.2 (12), tRNAscan-SE v1.3.1 (13), and Prodigal v2.6.3 (14) were used. The annotation was based on the homologs in “*Candidatus* Phytoplasma aurantifolia” NCHU2014 (CP040925-CP040926) (15) as identified by OrthoMCL v1.3 (16), followed by manual curation based on BlastKOALA v.2.2 (17) and GenBank (6). Putative secreted proteins were identified using SignalP v5.0 (18) and filtered by TMHMM v2.0 (19).

The assembly produced two circular contigs, including a 705,138 bp chromosome (26.7% G + C) and a 4,757 bp plasmid (23.4% G + C). The Illumina and ONT reads provided 107- and 115-fold coverage, respectively. The chromosomal and plasmid contig were rotated to have *dnaA* and a replication-associated gene as the first gene, respectively. The annotation contains 6 rRNA genes, 32 tRNA genes, 722 protein-coding genes, and 5 pseudogenes.

## Data Availability

This genome project has been deposited in NCBI under the accession number PRJNA939941. All raw reads derived from the infected plant sample have been deposited at the NCBI Sequence Read Archive under the accession numbers SRX19554650 and SRX19554651. The genome sequence has been deposited at GenBank under the accession numbers CP119306-CP119307.
